# Diffuse Large B-cell Lymphoma Occurring with Rhinophyma: A Case Report

**DOI:** 10.7759/cureus.2536

**Published:** 2018-04-25

**Authors:** Samuel Shatkin, Michael Shatkin, Katherine Smith, Leah E Beland, Adam J Oppenheimer

**Affiliations:** 1 Department of Plastic Surgery, Aesthetic Associates Centre, Buffalo, USA; 2 Department of Plastic & Reconstructive Surgery, Aesthetic Associates Centre, Buffalo, USA; 3 Medical Education, University of Central Florida College of Medicine, Orlando, USA; 4 Department of Plastic & Reconstructive Surgery, Oppenheimer Plastic Surgery, Orlando, USA

**Keywords:** rhinophyma, lymphoma, rosacea, diffuse large b-cell lymphoma, dlbcl, reconstruction, basal cell carcinoma

## Abstract

Rhinophyma is the final stage in the evolution of acne rosacea, a common vasoactive dermatosis. Individuals with rhinophyma present with a typical, disfiguring nasal appearance consisting of bulbous enlargement, erythema, and telangiectasia with a sebaceous, oily skin surface. This classic appearance permits a facile diagnosis but may also lead the physician to overlook a coexistent malignancy. We report the occurrence of a diffuse large B-cell lymphoma (DLBCL) arising synchronously with a marked rhinophyma. A wide local excision of the malignancy was performed, and the defect was reconstructed with forehead flaps. The rhinophyma was treated with a skin graft and cheek flaps. Following surgery, chemotherapy was used to manage the systemic disease. This case demonstrates the necessity for clinical scrutiny in the diagnosis and treatment of rhinophyma. It is imperative to entertain a high degree of suspicion when non-typical changes are observed within a rhinophymatous lesion or in adjacent areas of the nose.

## Introduction

Rhinophyma is the final stage of acne rosacea, a common vasoactive dermatosis [1]. Rhinophyma presents with a disfiguring nasal appearance consisting of bulbous enlargement, erythema, and telangiectasia with a sebaceous skin surface. Dermal and sebaceous gland hyperplasia, fibrosis, and sebum inspissation are the histological hallmarks of rhinophyma. Due to the indolent presentation and benign course, patients with these lesions are often followed without concern.

Malignancy should be considered when the lesion exhibits non-typical changes, including ulceration, drainage, and rapid growth. Sudden clinical changes within a longstanding rhinophyma should also alarm the physician [2]. Numerous case reports and clinical studies have elucidated the wide spectrum of malignancies that may coexist with rhinophyma. In a series of 47 patients with rhinophyma, Acker and Helwig reported five cases of basal cell carcinoma, one squamous cell carcinoma, and one sebaceous adenoma coexisting on the nose [3].

We present the case of a slow-growing rhinophyma with an adjacent cutaneous lymphoma that may have been overlooked based on its appearance as a benign rhinophymatous lesion. This case demonstrates the necessity for clinical scrutiny in the diagnosis and treatment of rhinophyma.

## Case presentation

A 69-year-old man presented with chief complaints of a growing mass on his nose, concomitant nasal airway obstruction, visual field impairment, and an inability to wear glasses. The patient also described the recent appearance of a glabellar lesion. Several weeks prior to our consultation, the patient sought the care of his primary care physician, who incised this newer lesion hoping to drain it. When this procedure was unsuccessful, the patient was referred for further treatment.

**Figure 1:**
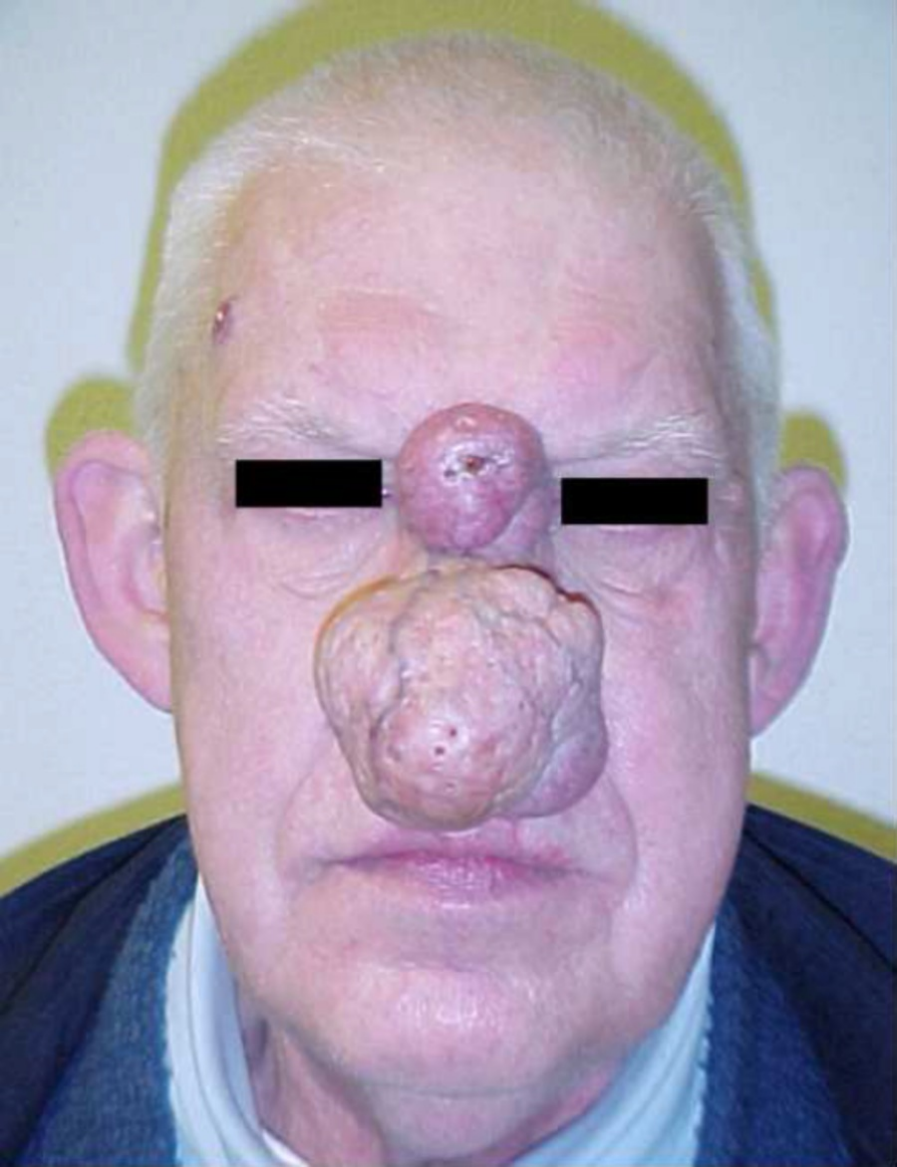
Frontal preoperative appearance

**Figure 2:**
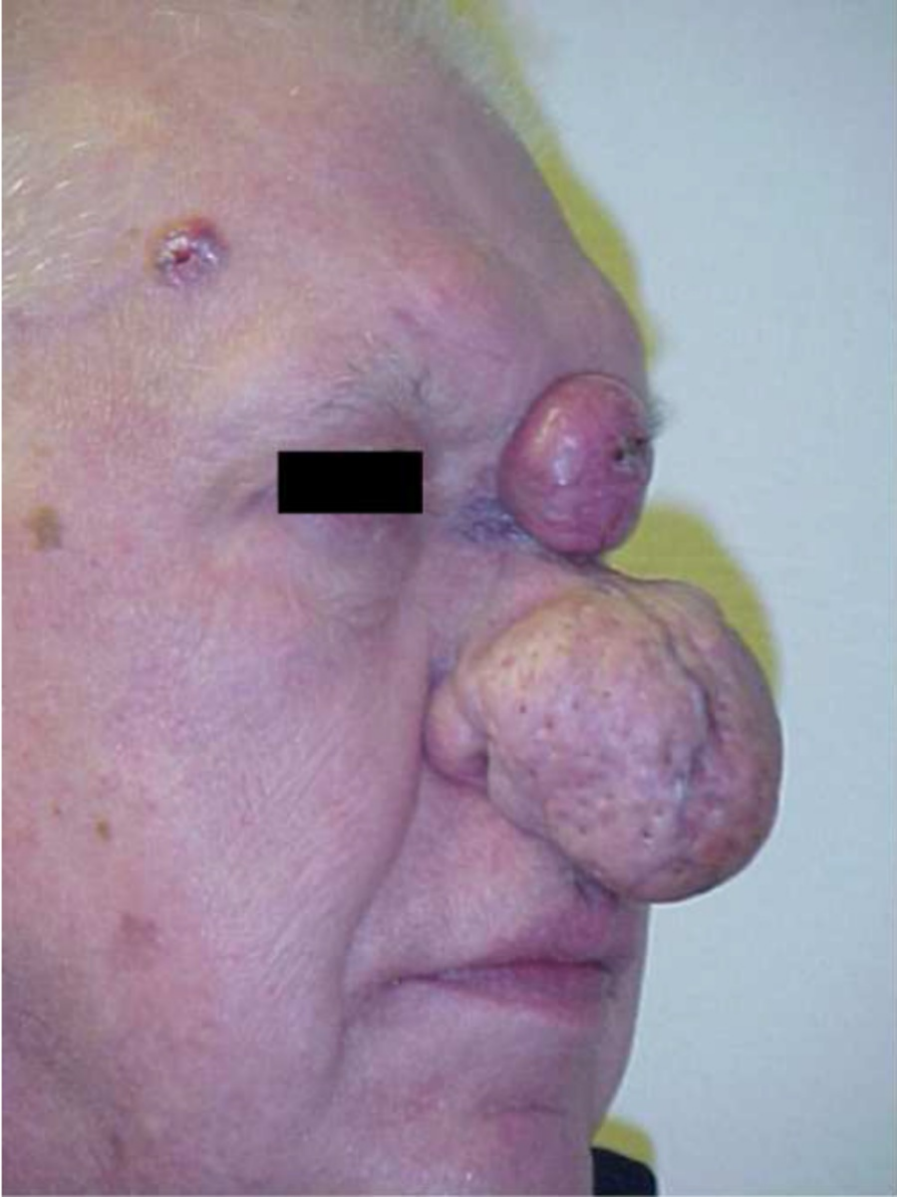
Preoperative appearance

Upon physical examination, the patient was found to have an extensive rhinophyma and additional lesions of the nasal glabella and right upper forehead regions (Figures 1, 2). The rhinophyma measured 6.7 centimeters (cm) in diameter. The patient reported that it had been enlarging for six years. Observation revealed an irregular, nodular tumor with telangiectasia and sebum inspissation. The glabellar lesion measured 3.0 cm in diameter and appeared as a discrete erythematous tumor with central ulceration and necrosis. The location of this lesion on non-sebaceous skin suggested a non-rhinophymatous lesion and raised our suspicion for malignancy. An additional 1.5 cm lesion of the right forehead region appeared as a round, telangiectatic nodule with a waxy border. A preoperative diagnosis of basal cell carcinoma for this separate lesion was confirmed by the pathological report.

**Figure 3:**
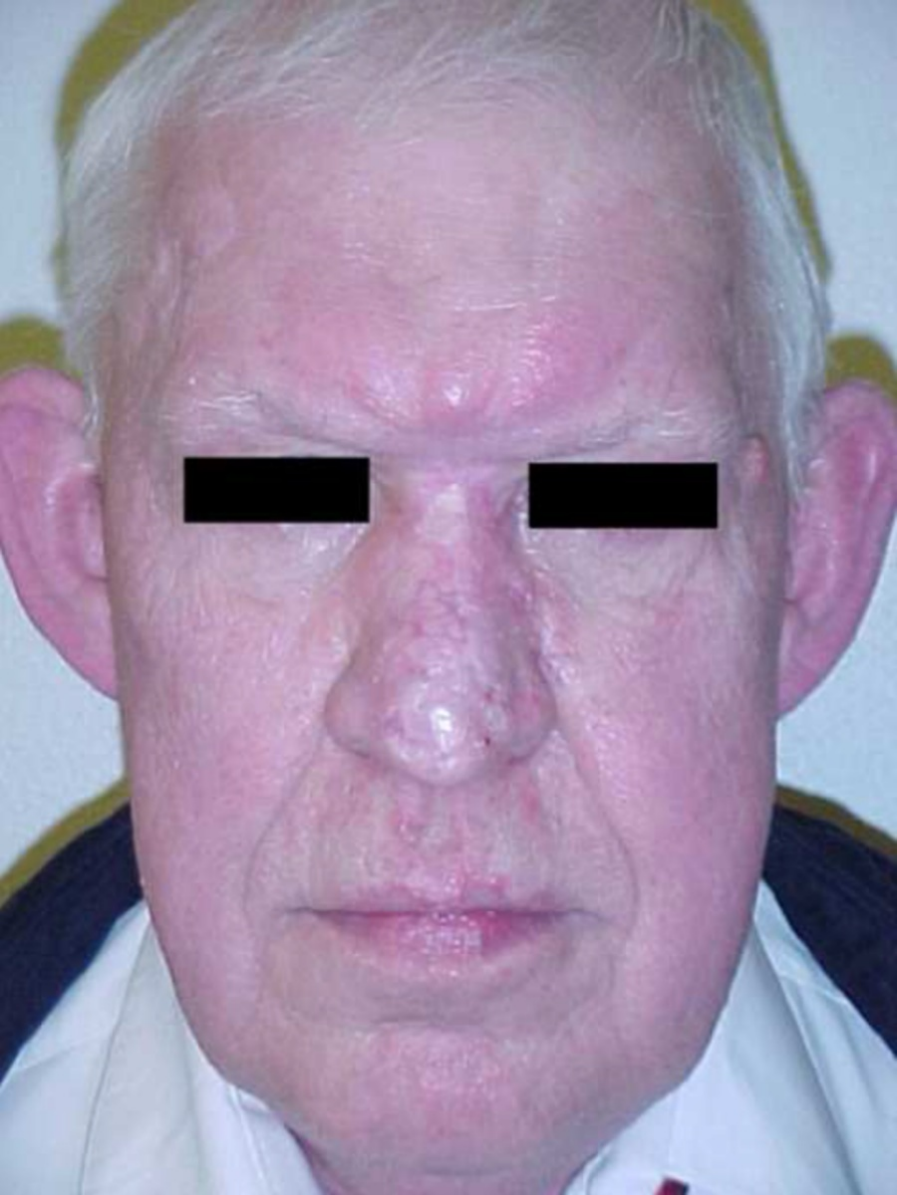
Frontal postoperative appearance

**Figure 4:**
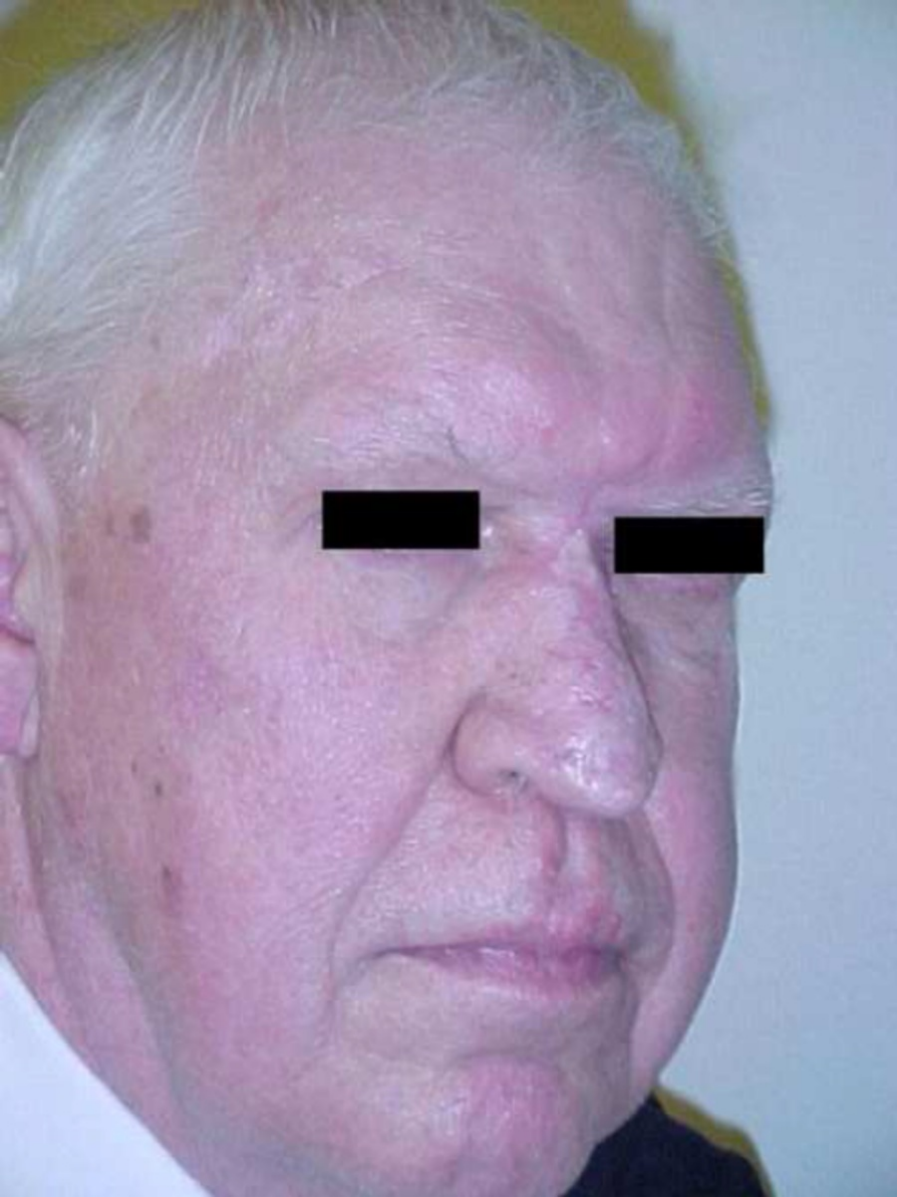
Postoperative appearance

Surgery was performed on the nasal lesions. The rhinophyma was excised and debulked, then closed with adjacent cheek flaps and skin graft. The glabellar lesion extended to the left medial canthal area. It was treated with wide local excision and was closed with forehead flaps. Frozen section analysis of the glabellar specimen revealed a diagnosis of diffuse large B-cell lymphoma. The area of rhinophyma on the lower portion of the nose displayed no evidence of lymphoma. After a three-week interval, the basal cell carcinoma of the right forehead was removed, and closure was accomplished with double opposing V-Y advancement flaps (Figures 3, 4).

The final pathology report confirmed the diagnosis of diffuse large B-cell lymphoma (DLBCL) with BCL6 positivity. The patient was referred to an oncologist for further treatment. Subsequent oncological workup revealed several positive lymph nodes in the anterior cervical chain. One such node was biopsied by a general surgeon and was found to be positive for diffuse large B-cell lymphoma. Intensive combination chemotherapy, including Rituxan®, Cytoxan®, Oncovin®, Adriamycin®, and prednisone, was initiated.

## Discussion

The causative relationship between acne rosacea and rhinophyma was first described by Virchow in 1846 [4]. While the precise mechanism for this evolution remains elusive, several theories have been proposed. The commonly accepted theory cites chronic inflammation, subsequent extracellular matrix degradation, and ultimate lymphatic failure progressing to fibroplasia as the underlying pathology in rhinophyma [5]. Alcohol is frequently cited as a causative agent, and terms, such as “rum blossom” and “whiskey nose”, permeate the layperson parlance. However, Curnier et al. found no difference in alcohol consumption between blepharoplasty patients and those undergoing surgical correction of rhinophyma [6].

Similarly, a causative relationship between rhinophyma and carcinoma has been proposed [3]. Acker and Helwig concluded that the incidence of basal cell carcinoma in patients with rhinophyma was higher than would be predicted by chance alone. They proposed that the increased cellular activity in rhinophymatous tissue was a premalignant condition that had a propensity to undergo carcinomatous degeneration. Subsequent reevaluation of their statistical methodology revealed that their data failed to reach significance at the 5% level [7]. The development of cutaneous lymphoma in our patient is likely coincidental. Regardless of causality, the coexistence of malignancy in our case merits discussion.

Cutaneous B-cell neoplasms mimicking rosacea and rhinophyma are rare. Stanway et al. reported the first case of a cutaneous B-cell lymphoma presenting as rhinophyma; the rhinophymatous lesion, in this case, existed concomitantly with nodules on the pinna and nailbeds [8]. Ogden and Coulson reported a case of a simultaneous T-cell and B-cell lymphoma presenting as rhinophyma [9]. More recently, Barzilai et al. published a case series of 12 patients with B-cell neoplasms mimicking rosacea or rhinophyma. Only four of their cases had pre-existing rosacea or rhinophyma [10]. None of these cases or case series described patients with diffuse large B-cell lymphoma presenting as rhinophyma. Although rare, these cases of malignancy existing within rhinophymatous lesions demonstrate that preoperative biopsy may be necessary in any case of rhinophyma with an atypical appearance, as a preoperative diagnosis may dictate the need for adjuvant chemotherapy.

Diffuse large B-cell lymphomas are aggressive neoplasms. Intensive combination chemotherapy, however, can induce complete remission of the disease in 60 - 80% of all patients. This depends on several prognostic factors, namely the degree of systemic dissemination and the immunologic properties of the B-cell lineage. In our patient, the tumor’s antigenic expression of the *BCL6* antigen indicated a favorable prognosis [11]. Our patient has successfully completed combination chemotherapy and is currently free of disease (Figures 3, 4).

## Conclusions

The classic appearance of rhinophyma permits a clinical diagnosis but may also lead the physician to overlook a coexistent malignancy. This case demonstrates the necessity for clinical scrutiny in the treatment of rhinophyma. Physicians must entertain a high degree of suspicion when non-typical changes are observed within a rhinophymatous lesion or in adjacent areas of the nose.
